# Modification of a COVID-19 Patient Testing Station With a Semiautomatic Sanitization System

**DOI:** 10.1093/annweh/wxac036

**Published:** 2022-05-21

**Authors:** Yan-Ren Lin, Nai-Rong Zhong, Cheng-Chieh Huang, Chih-Pei Su, Chien-Yu Shen, Lun-De Liao

**Affiliations:** Department of Emergency and Critical Care Medicine, Changhua Christian Hospital, Changhua 500, Taiwan; Department of Post-Baccalaureate Medicine, College of Medicine, National Chung Hsing University, Taichung 402, Taiwan; School of Medicine, Kaohsiung Medical University, Kaohsiung 807, Taiwan; School of Medicine, Chung Shan Medical University, Taichung 402, Taiwan; Institute of Biomedical Engineering and Nanomedicine, National Health Research Institutes, Zhunan Township, Miaoli County 35053, Taiwan; Department of Emergency and Critical Care Medicine, Changhua Christian Hospital, Changhua 500, Taiwan; Department of Biological Science and Technology, National Yang Ming Chiao Tung University, Hsinchu City 30010, Taiwan; Department of Emergency and Critical Care Medicine, Changhua Christian Hospital, Changhua 500, Taiwan; Institute of Biomedical Engineering and Nanomedicine, National Health Research Institutes, Zhunan Township, Miaoli County 35053, Taiwan; Institute of Biomedical Engineering and Nanomedicine, National Health Research Institutes, Zhunan Township, Miaoli County 35053, Taiwan

To the Editor:

Globally, many different types of stations were built to enable testing of patients for COVID-19 while preventing exposures of medical personnel. In Taiwan, these were called non-exposure testing stations, and were kiosks that separated personnel from patients but included fixed gloves through which personnel could collect samples (Lee, 2020).

Overall, there are two major limitations for operating an these testing stations. First, it is very difficult for medical personnel inside the stations to clean the outside zone by themselves (including the outside windows, gloves, tables, and chairs) without any additional assistance. Second, the sampling area in which the patient sits (or stands) might harbor some tiny fine droplets or aerosol particles that contain infectious virus, especially at those stations that do not have rigorous air circulation (i.e., inside a hospital or other buildings), and even outdoor sampling areas would need to be covered in the event of heavy rain or snow (Morawska *et al.*, 2020). Although highly atomized disinfectants would be helpful in reducing the abundance of viruses in aerosol particles and the environment (by increasing humidity, chemical structure damage, or the scavenging effect) (Anand, 2020; Mecenas *et al.*, 2020), they have not been widely applied in sampling stations.

To solve the two major limitations and save manpower, we have developed a simple outdoor semiautomatic sanitization system that can be controlled by medical staff remotely with a simple setting to trigger the disinfection control circuit. The control circuit has two control modes: a fixed time-triggered control mode and a manual continuous-triggered control mode, which controls the sanitization pump to dispense disinfectant and works with multiple disinfection nozzles set above the sampling station. All the physical layouts of this system are created with 3D printing to advance time-to-market turnaround. The spray particle size of this system can range from 3 to 7 µm with a high atomization degree. Therefore, the disinfectant can quickly (less than 1 s) and naturally dry after spraying, and its atomization range is designed to adequately cover the sampling zone (Fig. 1). In our observation test, we found that our system could cover 100% of the area of sample zone sanitization (in 10 s) compared to medical staff only (65%, 30 s) and staff and patient cooperation (92%, 90 s) (Fig. 2).

**Figure 1.**
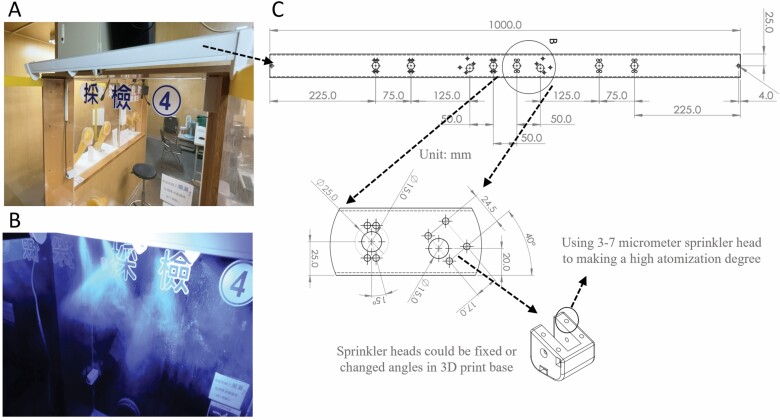
The system can cover 100% of the sampling area of zone sanitization with 10 s of spraying and provides a highly atomized design concept that might further sterilize the local air within the sampling zone. (A) The device can simply be hung (or placed on) the outside of the sampling zone. (B) The fluorescent spray particles can range from 3 to 7 µm with a high degree of atomization. (C) The original size of the system.

**Figure 2.**
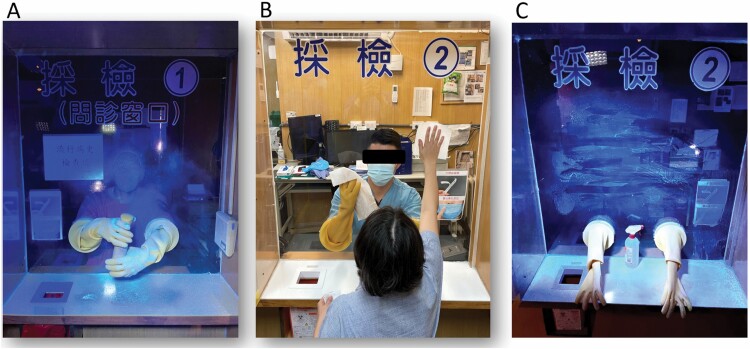
Measurement of the median percentages of the sanitized area and the time cost in the sampling zone using fluorescent substances with a 20 W UV light (365 nm). (A) Medical staff only (65% of the area sanitized, 30 s); (B) staff and patient cooperation; (C) the outcome of cooperation (92% of the area sanitized, 90 s). All tests were repeated 5 times. Areas with no fluorescent response (reflex) were considered not cleaned.

Notably, the 3D printing outdoor semiautomatic sanitization system is very easy to create and can even be made by the medical personnel. More importantly, it would be very useful in providing clean area for next patient, reducing concerns of the disease transmission.

## Data Availability

No data were used in this study.
